# Supplementary material for: Tradeoff between speed and robustness in primordium initiation mediated by auxin-CUC1 interaction
Source: Nat Commun. 2024 Jul 13;15:5911. doi: 10.1038/s41467-024-50172-9 (PMC11246466; doi:10.1038/s41467-024-50172-9)
Supplement: Supplementary file 3 — Description of additional supplementary files [file 41467_2024_50172_MOESM3_ESM.pdf]

<u>Description of Additional Supplementary Files</u>

Supplementary Data 1. Computational model of auxin pattern formation in the floral meristem. Also uploaded on GitHub: https://github.com/RoederLab/MassSpringAuxin

Supplementary Movie 1. *CUC1* expression pattern in WT vs *drmy1* buds. Related to Fig. 1.

Shown are *pCUC1::3xVENUS-N7* (yellow, top row) and VENUS merged with the Chlorophyll channel (magenta, bottom). Time points are 6 hours apart. Scale bar, 25 µm.

Supplementary Movie 2. CUC1 protein accumulation pattern in WT vs *drmy1* buds. Related to Fig. 1.

Shown are *pCUC1::CUC1-GFP* (yellow, top row) and GFP merged with the Chlorophyll channel (magenta, bottom). Time points are 6 hours apart. Scale bar, 25 µm.

Supplementary Movie 3. Auxin signaling pattern in a WT bud. Related to Fig. 4.

Shown are *DR5::3xVENUS-N7* (cyan, left) and DR5 merged with the Chlorophyll channel (magenta, right). Time points are normalized relative to the beginning of stage 2 (0 h) and are 6 hours apart. Scale bar, 25 µm. Same for Supplementary Movies 4-7.

Supplementary Movie 4. Auxin signaling pattern in a *5mCUC1* bud. Related to Fig. 4.

Supplementary Movie 5. Auxin signaling pattern in a *cuc1* bud. Related to Fig. 4.

Supplementary Movie 6. Auxin signaling pattern in a *drmy1* bud. Related to Fig. 4.

Supplementary Movie 7. Auxin signaling pattern in a *drmy1 cuc1* bud. Related to Fig. 4.

Supplementary Movie 8. Model of auxin pattern formation in WT, *cuc1*, *drmy1*, and *drmy1 cuc1* under moderate growth rate (0.8). Related to Fig. 6.

Supplementary Movie 9. Model of auxin pattern formation in WT and *drmy1* under low (0.4), intermediate (0.8), and high (1.2) growth rate. Related to Fig. 7.

Supplementary Movie 10. Model of auxin pattern formation in WT, *cuc1*, *drmy1*, and *drmy1 cuc1* under moderate growth rate (0.8), and WT and *drmy1* under reduced growth rate (0.4), when auxin noise is set constant (temporally unchanging). Related to Supplementary Fig. 8.
