# Supplementary figures and images for: Tradeoff between speed and robustness in primordium initiation mediated by auxin-CUC1 interaction
Source: Nat Commun. 2024 Jul 13;15:5911. doi: 10.1038/s41467-024-50172-9 (PMC11246466; doi:10.1038/s41467-024-50172-9)

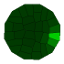

Supplement: Supplementary file 4 — Supplementary Data 1 [file 41467_2024_50172_MOESM4_ESM.zip › MassSpringAuxin/CellDisk.png]

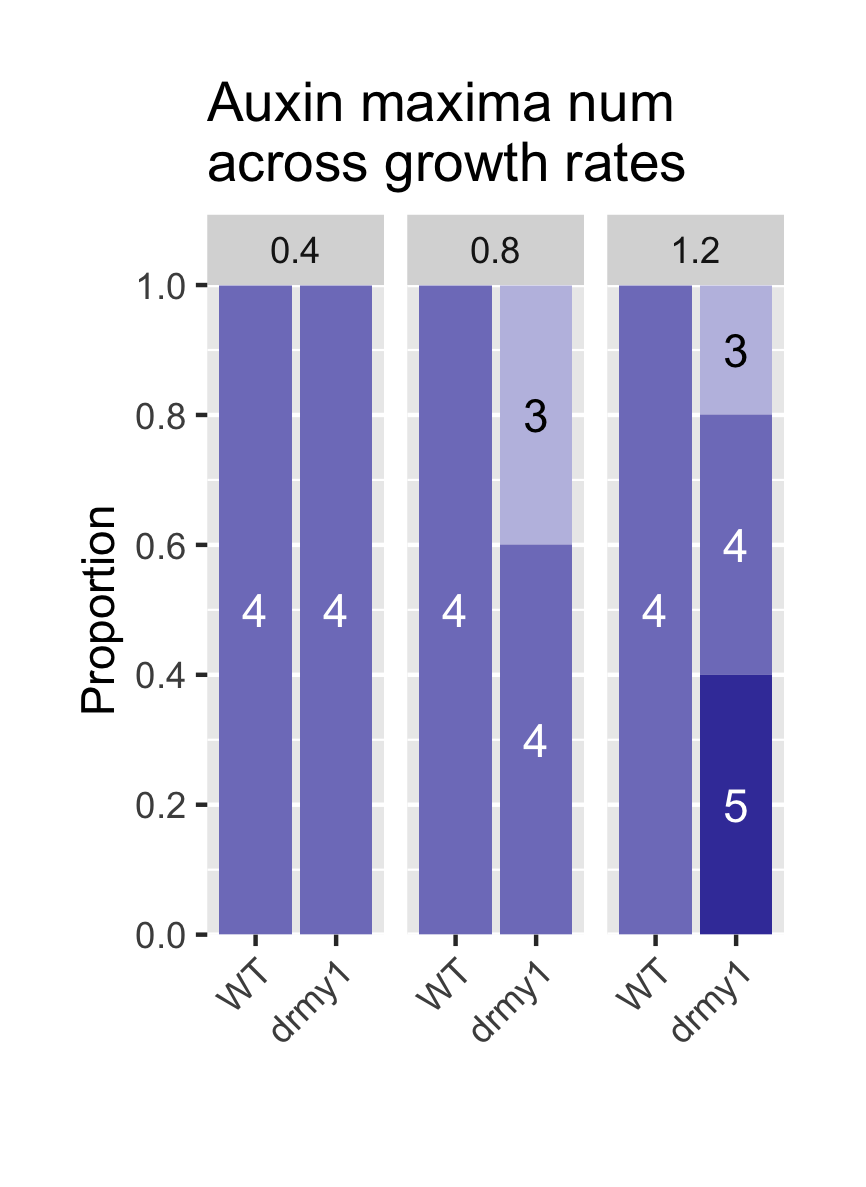

Supplement: Supplementary file 4 — Supplementary Data 1 [file 41467_2024_50172_MOESM4_ESM.zip › Plots/AuxMaxNum_WT-drmy1_growth-rates.png]

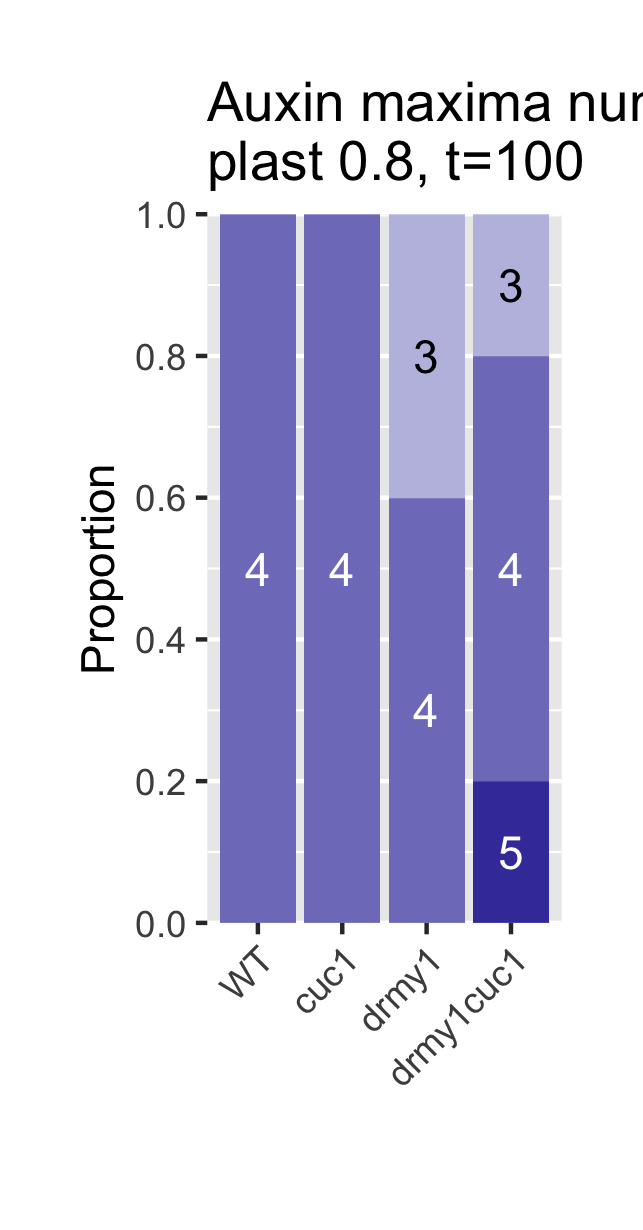

Supplement: Supplementary file 4 — Supplementary Data 1 [file 41467_2024_50172_MOESM4_ESM.zip › Plots/AuxMaxNum_plast0-8.png]

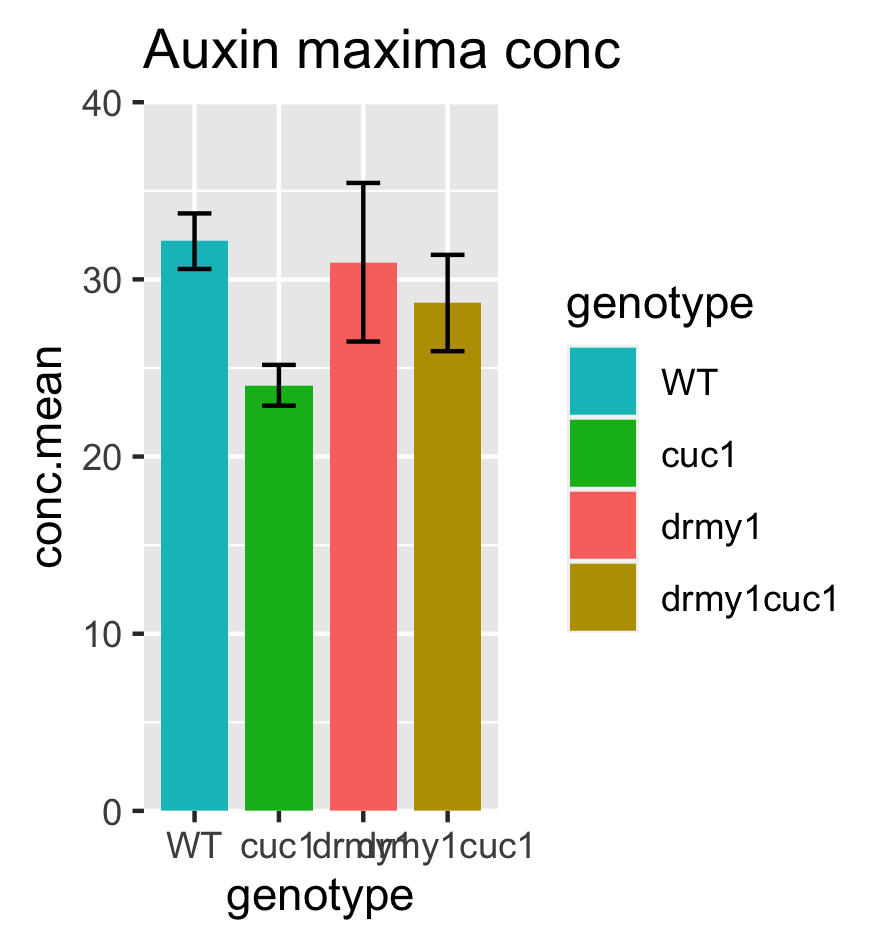

Supplement: Supplementary file 4 — Supplementary Data 1 [file 41467_2024_50172_MOESM4_ESM.zip › Plots/auxConc_plast_0-8_t100.png]
